# Post Traumatic Vesicocutaneous Fistula in the Thigh

**Published:** 2013-11-18

**Authors:** Vinod Priyadarshi, Rajkumar Singha Mahapatra, Dilip Kumar Pal, Anup Kumar Kundu

**Affiliations:** Department of Urology, Institute of Postgraduate Medical Education and Research, Kolkata. India

**Dear Sir,**

A vesicocutaneous fistula (VCF) secondary to trauma is a known entity. In children, bladder injuries are mostly intraperitoneal and therefore development of a VCF in the thigh is extremely rare. We report here one such VCF in the thigh following an extraperitoneal bladder injury.

A 12-year-old boy presented with fever, suprapubic pain, swelling and watery discharge from the repaired wound site at the right upper thigh. He had a history of fall from a tree and sustained penetrating injury to right thigh by a sharp wooden twig two days back. Primary treatment and repair of the wound was done by a local physician. Though he was able to void, he subsequently developed suprapubic swelling and pain with continuous watery discharge from the wound site. On examination, boy was febrile and lower abdomen was tender. There was 10 cm x 10 cm ill-defined swelling involving lower abdomen, right groin and inner thigh along with a 4 cm size stitched wound at about 7.5 cm below and lateral to the right pubic tubercle. From the inner corner of the wound, clear fluid was trickling intermittently. Ultrasonography (USG) revealed a huge (>200ml) extraperitoneal fluid collection in right groin and upper thigh extending up to the wound, however no foreign body visualized. Initial investigations showed leucocytosis (TLC- 16000/cmm) and raised serum creatinine (1.8 mg/dl), while urine analysis revealed RBCs and the urine culture grew no bacteria. Gentle urethral catheterization was done and subsequently abdominal swelling disappeared. Trickling fluid became bluish when methylene blue was instilled into the bladder and catheter clamped. This suggested a communication between bladder and the thigh wound. All stitches were then removed, and debridement of the wound done. The diagnosis of fistula was further confirmed by doing stress cystogram which revealed leakage of contrast outside the bladder into the pelvis and upper thigh (Fig. 1). Within 24 hours, groin swelling also subsided and wound became dry. A review USG after four days revealed no collection. He was discharged on fifth day with normal serum creatinine (1.2 mg/dl) with urethral catheter which was removed in 3rd week after complete healing of the thigh wound. Review USG and micturating cystourethrogram (MCUG) done were normal.

**Figure:**
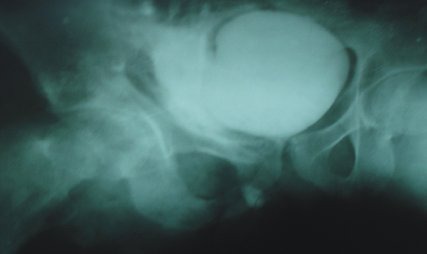
Figure 1: MCUG showing leakage of contrast outside the bladder in the pelvis and upper thigh.

Although, persistence of longstanding suprapubic cystostomy is the most common cause of VCF in routine urological practice[1], it may occur due to pelvic trauma, infection, neoplasm, bladder calculus, and bladder diverticulum or following surgical procedures as open prostatectomy, pelvic irradiation and total hip arthroplasty.[2-5] A VCF at a distant location has been reported in hypogastrium, perineum and scrotum. A case report of VCF in thigh that developed 7 years after a pelvic injury is found on literature search.[6] Our case is unique one, as it developed due to a penetrating wound in thigh after fall from height leading to extraperitoneal bladder rupture. This is well established that all cases of extraperitoneal bladder injury should be treated with adequate bladder drainage which should be done at the earliest.[2] In the initial period, raised creatinine level may reflect urinary absorption from extraperitoneal collection site.

MCUG is sufficient to diagnose an uncomplicated fistula but other cross-sectional imaging such as computed tomography (CT) scan and Magnetic Resonance Imaging (MRI) are needed if the fistulous tract is complicated.[7] A urinary diversion and appropriate wound care lead to spontaneous closure of the fistula in most of the cases. For non-healing and chronic cases, fistulectomy or even partial cystectomy has been described.[5,6]

## Footnotes

**Source of Support:** Nil

**Conflict of Interest:** None declared

